# Multi-energy conversion based on game theory in the industrial interconnection

**DOI:** 10.1371/journal.pone.0245622

**Published:** 2021-01-19

**Authors:** Jianjia He, Xiumeng Wu, Junxiang Li, Shengxue He

**Affiliations:** 1 Business School, University of Shanghai for Science and Technology, Shanghai, China; 2 Super Network Research Center (China), Shanghai, China; Chongqing Jiaotong University, CHINA

## Abstract

The multi-energy conversion system (MCS) plays an important role in improving the utilization of energy resources and realizing the energy transition. With the application of the new generation of information technologies, the new MCS can realize real-time information interaction, multi-energy collaboration, and real-time demand response, in which energy suppliers can intelligently motivate consumers' energy use behavior. In this paper, an MCS coupled with a cloud platform is proposed to address information explosion and data security issues. Due to the development of Internet technology, the increasing energy data, and the serious energy coupling, it is difficult for traditional optimization methods to deal with the interaction between participants of the MCS. Therefore, the non-cooperative game is used to formulate the interactions between participants with the aim of maximizing the energy suppliers' profit and minimizing the customers' cost. It is proved that the game model is an ordinary game with one Nash equilibrium. The simulation was performed with a gradient projection algorithm and the results show that the proposed MCS improves energy utilization efficiency through energy conversion while ensuring consumer satisfaction, and benefits both the customers and suppliers by reducing the energy consumption cost and the peak load demand, which effectively improve the supply quality and enrich the energy consumption patterns.

## Introduction

In recent years, the rapid economic development in China has stimulated a shift in energy consumption patterns, which embodies the amount of energy consumption as well as diversity [1]. The total energy consumption in China has been on the rise for the past 20 years. According to the National Bureau of Statistics, it reached 4.8 billion tons of standard coal in 2019, which is approximately 2.3 times more than in 2000. At present, the scale of renewable energy development and construction represented by hydropower, wind power, and solar energy is gradually expanding, but non-renewable energy still dominates the energy consumption market in China [2]. Therefore, it is a challenge to strengthen energy interconnection, improve energy utilization efficiency, change energy structure, and finally realize energy transformation [3]. Among different techniques for energy management, the multi-energy conversion system (MCS) is one of the effective approaches that can weaken the dependence on fossil energy. This system operates with a conversion device (such as heat pumps [4], gas boilers [5], and gas-fired combined heat and power plants(CHP) [6] to convert excess generated energy effectively into other forms of energy, thereby, improving the utilization of energy resources [7]). In addition, advanced metering infrastructure and intelligent instruments in the system enable energy suppliers to provide customers with historical energy consumption records and real-time price data [8], effectively improving the efficiency of energy generating and monitoring [9, 10].

With the application of cloud computing, the internet of things, big data, artificial intelligence, and other new-generation information technology, the communication and cooperation between energy-related companies and consumers have become more convenient, leading to closer interconnections between the energy industry and other industries. Increasingly, energy enterprises are eager to explore the “Internet plus” business model of the whole industrial chain. In fact, industrial interconnection provides an effective solution to the growing demand for energy consumers through energy interconnection and conversion [11]. A third-party platform called a cloud platform can be used to integrate information to enhance the liquidity and collaboration between energy companies and consumers. Cloud platforms are skilled at processing mass data with high efficiency [12] and can automatically record and calculate energy performance through uploaded energy consumption data. By directly observing energy performance fluctuations, users monitor or modify the operating performance of equipment or systems in real-time, which not only reduces the chance of abnormal performance detection but also helps maintain good energy performance. For instance, Tseng and Lee et al. [13] integrated Internet communications, cloud computing technology, and cloud energy management services to reduce the energy consumption of small and medium-sized enterprises. Sun and Li et al. [14] used the efficient virtualization of the ontology modeling technique to build models to improve resource utilization. With the application of the cloud platform, enterprises can simply make a request to the platform rather than rely on data planning to obtain the cleaned data [15]. In addition, through the combination of encryption technology and Web Service technology, data security in the process of platform and client transmission is guaranteed. Thus, the cloud platform can help monitor energy usage more efficiently and integrate various energy systems more intelligently [16, 17].

As for the studies of MCS, a number of recent studies have addressed multi-energy integration and energy conversion. Some researchers use different mathematics methods, such as programming models, graph theory, and advanced algorithms, to acquire the optimal overall system scheme [18–20]. Based on the design and operation of energy conversion systems, the concept of distributed energy technology has been applied to the aspects of energy production, storage, and distribution in an MCS [8, 21, 22]. In general, energy conversion efficiency, economy, and environmental impact are often evaluated with optimization models to examine the overall quality of an MCS [23–25]. Besides, Chen established a nonlinear model to distinguish the relationships between conversion efficiency and government asymmetric information [26]. These studies above provide a useful method for the system design and solution of the optimal operation. However, conventional energy-management researches focus on the optimization of system performance and advanced algorithms, ignoring the interaction between energy suppliers and consumers and its impact on the operational decisions of the system.

During the operation of the MCS, suppliers and consumers continuously change their strategies to obtain more profit, and tend to interact with each other in the energy interaction process. In fact, scholars mainly use game theory to explore the competitive and interactive behavior among different participators [27, 28]. Chen studied the problems of bidding and market equilibrium under the coexistence of multiple stakeholders in a multi-energy system [11]. A Stackelberg game model based on energy management is established to determine the optimal scheduling scheme of bi-directional energy demands conversion, aiming at optimizing the daily peak load shifting [29]. Liu modeled the interaction between multiple users as a non-cooperative congestion game and proved the existence of the Nash equilibrium point [30]. It can be concluded that non-cooperative game theory is used to analyze the decision-making process of stakeholders in the MCS with partially or completely conflicting goals. Each participant tries to maximize the profit that does not only depend on its choices but also on the decisions of other stakeholders, leading to the reduction of the overall interests. However, the realization of energy conversion has changed the way to satisfy one consumer's energy demand while reducing the supply of other consumers. The application of energy conversion devices and new technologies have greatly improved the quality of energy supply and enriched energy consumption patterns. As the collaboration greatly improves operational efficiency [31, 32], the competition under industrial interconnection not only promotes the development of individuals but also achieves the goal of mutual benefit and win-win. In the potential game, the utility function of each player is consistent with the potential function, which ensures the consistency of the individual and the whole and exactly conforms to this special competition. Therefore, this paper uses Stackelberg game theory and potential game theory to construct competitive interaction models between suppliers and consumers and different consumers. Energy supply companies motivate and mobilize consumers' behavior by providing real-time energy price information, that is, energy suppliers are leaders and consumers are followers. Besides, the mathematical solution of game theory faces a common problem: it is necessary to prove the existence of the equilibrium solution of the game, and the process of solving the equilibrium point is usually cumbersome. As one of the non-cooperative game, potential game guarantees the existence of Nash equilibrium solution without tedious verification of equilibrium solution and has advantages in application analysis.

In this paper, game theory was applied to multi-energy conversion based on cloud platform under industrial interconnections. The main contents of this paper can be summarized as follows. First, we propose a multi-energy conversion program between energy companies and consumers, which is proved as a Stackelberg game with one leader and multi-followers. The energy company is the only leader and decides the energy prices. The followers are the consumers who will adjust their energy needs accordingly. Secondly, we prove that the competition among consumers is an ordinal potential game with one Nash equilibrium. Thirdly, we developed a gradient projection algorithm to measure the system. Lastly, the simulation results showed that energy can converse more efficiently and both energy companies and customers could decrease their payments in the program.

The remainder of this paper is organized as follows. In Section 2, we specify the components of the multi-energy conversion system and the general flow of the system operation. The energy conversion between energy suppliers and consumers is modeled and described in detail. In Section 3, game analysis is introduced and the uniqueness of the Nash equilibrium is proven. A gradient projection algorithm is also proposed to find the Nash equilibrium. In Section 4, we present the results and analysis of the simulations. The convergence of the proposed algorithm is also discussed. Finally, Section 5 provides the research conclusions.

## System model

**Fig 1:**
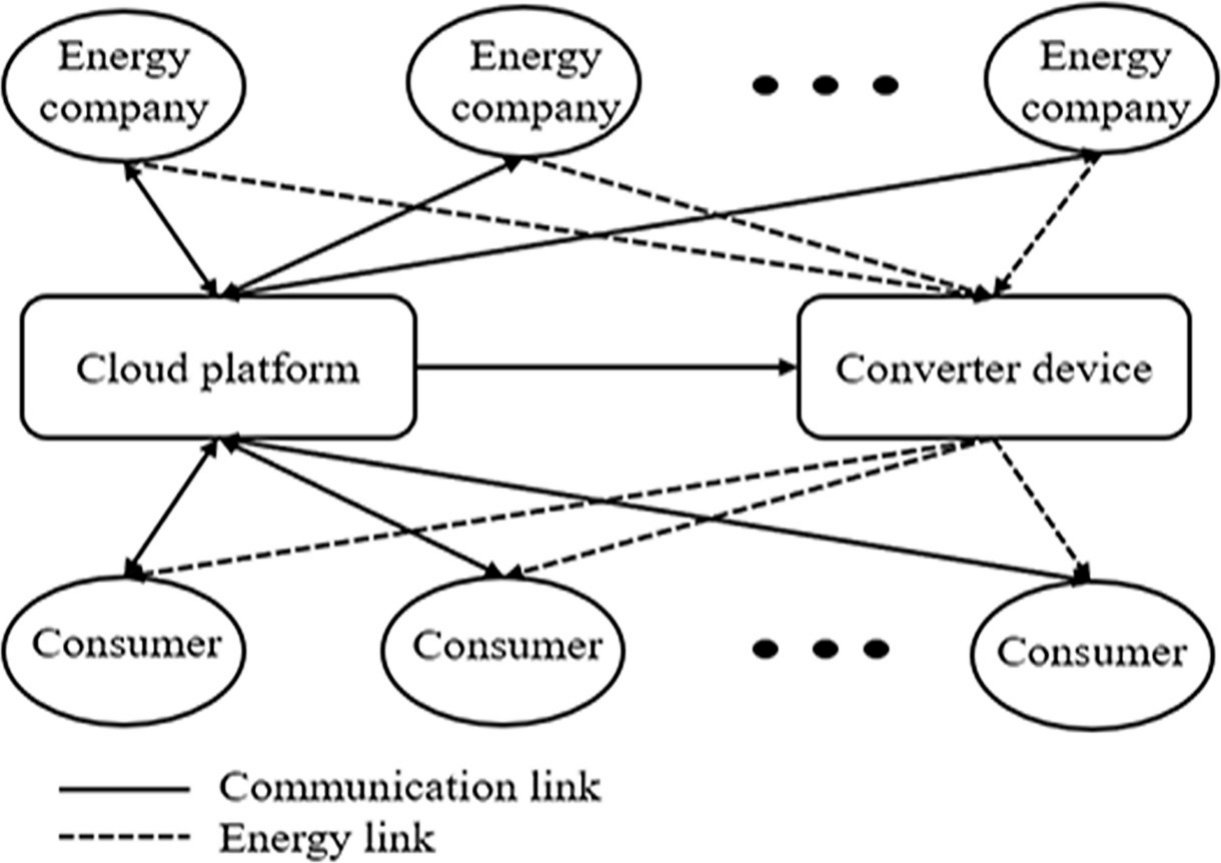
Diagram of a multi-energy conversion system (MCS) consisting of energy companies, consumers, the cloud platform, and the converter device.

In this paper, the energy company and consumers share information with each other through the cloud platform in a multi-energy conversion system, where different consumers and energy suppliers are controlled by this communication. The cloud platform is based on the combination of hardware resources and software resources, through the cloud for data storage, computing, and network communication. Therefore, the supply information of energy suppliers, the historical consumption data of consumers, and the amount of energy conversion are all stored and updated constantly in the cloud large-capacity service system. In this MCS, the consumers tend to shift the energy consumption style to get more convenience during peak hours. The cloud platform allocates and releases information resources in real-time according to the actual situation of users, and then sends adjustment signals to the converter devices to optimize the supply of the energy company intelligently. Fig 1 depicts the interactions between the energy and consumers who communicate through the cloud platform. In fact, thermal, electric, and chemical are the three basic forms of energy [33]. Hence, electricity, natural gas, and heat power are the representatives of these three basic energies in this paper.

In the MCS, gas, electricity, and heat power are supplied by energy companies and will be converted, conditioned, and stored through the converter device. Let *E*^*out*^, *G*^*out*^, and *H*^*out*^ denote the output of electricity, gas, and heating power in the converter device, respectively. Let *E*^*in*^, *G*^*in*^, and *H*^*in*^ denote the input electricity, gas, and heating powers in the converter device, respectively, which are also provided by energy companies. In an energy conversion station, the amount of energy input and output is correlated according to the following equation:

$$\left[\begin{array}{c} E^{out}\\ G^{out}\\ H^{out} \end{array}\right]=\left[\begin{array}{ccc} 1-\lambda_{1} & \lambda_{2}\eta_{ge} & 0\\ 0 & 1-\lambda_{2}-\lambda_{3} & 0\\ \lambda_{1}\eta_{eh} & \lambda_{3}\eta_{gh} & 1 \end{array}\right]\left[\begin{array}{c} E^{in}\\ G^{in}\\ H^{in} \end{array}\right]$$ (1)

The electricity transforms to heat power in the system with efficiencies denoted by *η*_*eh*_. The gas transforms to electricity and heat power in the system with efficiencies denoted by *η*_*ge*_ and *η*_*gh*_, respectively. *λ*_1_, *λ*_2_ and *λ*_3_∈[0,1] are the dispatch factors of different energy. They define the dispatch of the electricity, gas, and heating powers provided to consumers through the converter device.

### Energy suppliers

On the supplier side, energy companies generate energy and send it to the converter device. In this study, we assume N energy consumers are served by one energy company in the MCS. The set of consumers is denoted by *n*∈{1,2,⋯,*N*}. One day is divided into T equal time slots. The set of time slots is denoted by *t*∈{1,2,⋯,*T*}. $E_{t}^{Total}$, $G_{t}^{Total}$, and $H_{t}^{Total}$ are the total amounts of electricity, gas and heat provided by energy companies at time *t*. $E_{n,t}^{in}$, $G_{n,t}^{in}$, and $H_{n,t}^{in}$ are the electricity, gas, and heat power provide to the consumer n before conversion at time *t*. Thus,

$$I_{t}^{Total}=\sum_{n\in N}I_{n,t}^{in},I=\{E,G,H\}$$ (2)

The electricity, gas, and heat costs are described by the commonly polynomial cost functions [34]: $c(E_{t}^{Total})=(\theta_{2}/2){\left(E_{t}^{Total}\right)}^{2}+\theta_{1}E_{n,t}^{in}+\theta_{0}$, $c(G_{t}^{Total})=(\vartheta_{2}/2){\left(G_{t}^{Total}\right)}^{2}+\vartheta_{1}G_{n,t}^{in}+\vartheta_{0}$, $c(H_{t}^{Total})=(\varphi_{2}/2){\left(H_{t}^{Total}\right)}^{2}+\varphi_{1}H_{n,t}^{in}+\varphi_{0}$, where *θ*_0_, *θ*_1_, *θ*_2_ *ϑ*_0_, *ϑ*_1_, *ϑ*_2_, *φ*_0_, *φ*_1_ and *φ*_2_ are the positive coefficients. This cost function is strictly convex and has positive and increasing derivatives. The energy company aims to supply amount of energy at a certain price to consumers to maximize its profit. The optimization problem of an energy company is defined as:

$$\begin{array}{l} \underset{U(I_{t}^{Total})}{\max}=\sum_{t\in T}\left[E_{t}^{Total}p_{e}(E_{t}^{Total})+G_{t}^{Total}p_{g}(G_{t}^{Total})+H_{t}^{Total}p_{h}(H_{t}^{Total})-c(E_{t}^{Total})-c_{g}(G_{t}^{Total})-c_{g}(H_{t}^{Total})\right]\\ s.t\;Equation(2) \end{array}$$ (3)

Where $I_{t}^{Total}=\{E_{t}^{Total},G_{t}^{Total},H_{t}^{Total}\}$. By introducing the Lagrange multiplier *α*_1_,*α*_2_,*α*_3_, the solution of the above optimization problem can be obtained as follows:

$$p_{e}(E_{t}^{Total})‐c^{\prime }(E_{t}^{Total})‐\alpha_{1}=0$$ (4A)

$$p_{g}(G_{t}^{Total})‐c^{\prime }(G_{t}^{Total})‐\alpha_{2}=0$$ (4B)

$$p_{h}(H_{t}^{Total})‐c^{\prime }(H_{t}^{Total})‐\alpha_{3}=0$$ (4C)

Where *c*′ is the derivative of *c*. Substituting the cost functions into Eq (4A)–(4C), the relationship between the energy price and the supply is obtained. Let $p_{e}(E_{n,t}^{in})$, $p_{g}(G_{n,t}^{in})$, and $p_{h}(H_{n,t}^{in})$ denote the price of electricity, gas, and heat power input in the conversion device at time t. In this paper, we assume that the conversion of energy will not lead to price changes. The equations (5A)–(5C) can be obtained according to the price invariance. Thus,

$$p_{e}(E_{n,t}^{in})=p_{e}(E_{t}^{Total})=\theta_{2}E_{t}^{Total}+\theta$$ (5A)

$$p_{g}(G_{n,t}^{in})=p_{g}(G_{t}^{Total})=\vartheta_{2}G_{t}^{Total}+\vartheta$$ (5B)

$$p_{h}(H_{n,t}^{in})=p_{h}(H_{t}^{Total})=\varphi_{2}H_{t}^{Total}+\varphi$$ (5C)

where *θ*, *φ*, and *φ* are positive coefficients. Eq (5A)–(5C) implies that the prices set by the energy company are equal to the marginal production costs.

### Energy consumers

The customer can obtain real-time electricity, gas, and heat prices through the cloud platform. During critical peak periods, the price of energy is higher and customers would not reduce the consumption of electricity, gas, or heat power. Thus, they will shift energy consumption. This means that consumers send a conversion signal to the platform, and then the platform’s direct converter device transforms the energy to meet the consumer needs.

$E_{n,t}^{out}$, $G_{n,t}^{out}$, and $H_{n,t}^{out}$ are the outputs of electricity, gas, and heat power in the converter device, respectively. $E_{n}^{d}$, $G_{n}^{d}$, and $H_{n}^{d}$ denote the electricity, gas, and heat demand for consumer n, respectively. Hence,

$$I_{n}^{d}=\sum_{n\in N}I_{n,t}^{out},(I=\{E,G,H\})$$ (6)

In the system, the electricity, gas, and heat output from the conversion device will be converted according to the needs of consumers, but the total energy consumption provided by the energy company does not change. That is, the total energy input is fixed for a period of time, while the total output is uncertain.

In the MCS, the consumers can distinguish the energy shortage by the price values. Game theory is a great method to analyze consumers. In the system, consumers are the players, and the strategy profile for consumer n is

$$x_{n}=\left\{E_{n,t}^{in},G_{n,t}^{in},H_{n,t}^{in}\right\},t\in \left\{1,2,\cdots ,T\right\}.$$ (7)

Thus, the payoff function of consumer n is defined as,

$$u_{n}(x_{n},x_{-n})=\sum_{I}\sum_{t\in T}\left(U_{n}^{i}(I_{n,t}^{out})-p_{t}^{i}I_{n,t}^{in}\right)$$ (8)

where *x*_−*n*_ = (*x*_1_,⋯,*x*_*n*−1_,*x*_*n*+1_,⋯*x*_*N*_), *I* = {*E*,*G*,*H*}.

In payment function (8), the terms of $U_{n}^{i}(I^{out})$ are the utility obtained by the customers, which is formulated as follows:

$$u_{n}^{i}(I^{out})=w_{n}^{i}I^{out}-(\alpha_{n}^{i}/2){\left(I^{out}\right)}^{2}$$ (9)

where, *w* and *α* are the parameters of the satisfaction equation. Clearly, the satisfaction functions are continuously differentiable and strictly concave, which is easy to prove.

Let $x_{n}^{*}$ denotes the strategy profile of energy consumer n in the Nash equilibrium.

$$\begin{array}{l} x_{n}^{*}\in \arg\;\max u_{n}(x_{n}^{*},x_{-n}^{*})\\ s.t.\;Equation(9) \end{array}$$ (10)

The price anticipating consumers know that the price of electricity, gas, and heat is calculated according to (2). Substituting (2) into (5), $x_{n}^{*}$ is the solution to the following optimization problem:

$$\begin{array}{l} \underset{y_{n}}{\max}=\sum_{t\in T}\sum_{n\in N}\left(U_{n}^{e}(E_{n,t}^{out})-(\theta_{2}E_{n,t}^{in}+\theta_{1})E_{n,t}^{in}\right)+\\ \;\sum_{t\in T}\sum_{n\in N}\left(U_{n}^{g}(G_{n,t}^{out})-(\vartheta_{2}G_{n,t}^{in}+\vartheta_{1})G_{n,t}^{in}\right)+\\ \;\sum_{t\in T}\sum_{n\in N}\left(U_{n}^{h}(H_{n,t}^{out})-(\varphi_{2}H_{n,t}^{in}+\varphi_{1})H_{n,t}^{in}\right)\\ s.t.\;Equation(6)and(9) \end{array}$$ (11)

The relationship between the energy input and output is as Eq (1). The relationship between *E*^*in*^, *G*^*in*^, and *H*^*in*^; and *E*^*out*^, *G*^*out*^, and *H*^*out*^ is given by inverting the matrix. Hence,

$$\left[\begin{array}{c} E_{n,t}^{in}\\ G_{n,t}^{in}\\ H_{n,t}^{in} \end{array}\right]=\left[\begin{array}{ccc} A_{n,t} & B_{n,t} & 0\\ 0 & C_{n,t} & 0\\ D_{n,t} & F_{n,t} & G_{n,t} \end{array}\right]\left[\begin{array}{c} E_{n,t}^{out}\\ G_{n,t}^{out}\\ H_{n,t}^{out} \end{array}\right]$$ (12)

where, $A_{n,t}=\frac{1}{1-\lambda_{1,n,t}}$; $B_{n,t}=\frac{-\lambda_{2,n,t}\eta_{ge}}{(1-\lambda_{1,n,t})\lambda_{n,t}}$; $C_{n,t}=\frac{1}{\lambda_{n,t}}$; $D_{n,t}=\frac{-\lambda_{1,n,t}\eta_{e,h}}{1-\lambda_{1,n,t}}$; $F_{n,t}=\frac{\lambda_{1,n,t}\lambda_{2,n,t}\eta_{eh}(\lambda_{1,n,t}\eta_{eh}\eta_{ge}‐\lambda \eta_{gh})}{\lambda_{n,t}\lambda_{1,n,t}\eta_{eh}(1-\lambda_{1,n,t})}$; $G_{n,t}=\frac{\lambda_{1,n,t}\eta_{eh}}{\lambda_{2,n,t}\eta_{gh}}$; *λ* = 1−*λ*_2_−*λ*_3_.

Thus, *x*_*n*_ will be converted to the strategy profile *y*_*n*_ correspondingly, which is defined as follows:

$$y_{n}=\left\{\lambda_{1,n},\lambda_{2,n},\lambda_{3,n},E_{n}^{out},G_{n}^{out},H_{n}^{out}\right\}$$ (13)

where, *λ*_1,*n*_ = {*λ*_1,*n*,*t*_}; *λ*_2,*n*_ = {*λ*_2,*n*,*t*_}; *λ*_3,*n*_ = {*λ*_3,*n*,*t*_}; $E_{n}^{out}=\{E_{n,t}^{out}\}$; $G_{n}^{out}=\{G_{n,t}^{out}\}$; $H_{n}^{out}=\{H_{n,t}^{out}\}$. *y*_*n*_ is the solution to the following optimization problem:

$$\begin{array}{l} \underset{y_{n}}{\max}=\sum_{t\in T}\sum_{n\in N}U_{n}^{e}(E_{n,t}^{out})-(\theta_{2}(A_{n,t}E_{n,t}^{out}+B_{n,t}G_{n,t}^{out})\\ \;+\theta_{1})(A_{n,t}E_{n,t}^{out}+B_{n,t}G_{n,t}^{out})+\sum_{t\in T}\sum_{n\in N}U_{n}^{g}(G_{n,t}^{out})\\ \;-(\vartheta_{2}C_{n,t}G_{n,t}^{out}+\vartheta_{1})+\sum_{t\in T}\sum_{n\in N}U_{n}^{h}(H_{n,t}^{out})-\\ \;\left(\varphi_{2}(D_{n,t}E_{n,t}^{out}+F_{n,t}G_{n,t}^{out}+G_{n,t}H_{n,t}^{out})+\varphi_{1}\right)\\ \;\left(D_{n,t}E_{n,t}^{out}+F_{n,t}G_{n,t}^{out}+G_{n,t}H_{n,t}^{out}\right)\\ s.t.\;Equation(6)-(10) \end{array}$$ (14)

The aim of each energy consumer is to determine its strategy profile *y*_*n*_ in Eq (14). In the meantime, other consumers' strategies are unchanged. Once all energy consumers find the optimal strategy profiles, the Nash equilibrium is determined.

## Stackelberg game

### Potential game among energy consumers

When the energy prices are given, consumers can adjust their consumption strategies to compete and maximize their profit. Here, the interaction among the consumers is an ordinal potential game. A game is an ordinal potential game if the ordinal function *P*(*y*) maintains $y=(y_{n}-y_{n-1}),\overset{⌢}{y}=({\overset{⌢}{y}}_{n}-{\overset{⌢}{y}}_{n-1})$. Thus

$$\begin{array}{l} U_{n}(y_{n}-y_{-n})-U_{n}({\overset{⌢}{y}}_{n}-{\overset{⌢}{y}}_{-n})\geq 0\\ \Rightarrow P_{n}(y_{n}-y_{-n})-P_{n}({\overset{⌢}{y}}_{n}-{\overset{⌢}{y}}_{-n})\geq 0 \end{array}$$ (15)

The left side of inequality (12) is equivalent to

$$\sum_{I}\sum_{t\in T}\left(U_{n}^{i}({\overset{⌢}{I}}_{n}^{out})-U_{n}^{i}(I_{n}^{out}\right))\geq \sum_{t\in T}\left(D_{1,t}+D_{2,t}+D_{3,t}-D_{4,t}-D_{5,t}-D_{6,t}\right)$$ (16)

where, $D_{1,t}=(A_{n,t}{\overset{⌢}{E}}_{n,t}^{out}+B_{n,t}{\overset{⌢}{G}}_{n,t}^{out})p_{e}(\theta_{1}(A_{n,t}E_{n,t}^{out}+B_{n,t}G_{n,t}^{out})+\theta_{0})$; $D_{2,t}=C_{n,t}{\overset{⌢}{G}}_{n,t}^{out}p_{g}(\vartheta_{1}C_{n,t}G_{n,t}^{out}+\vartheta_{0})$; $D_{3,t}=(D_{n,t}{\overset{⌢}{E}}_{n,t}^{out}+F_{n,t}{\overset{⌢}{G}}_{n,t}^{out}+G_{n,t}{\overset{⌢}{H}}_{n,t}^{out})p_{h}(\varphi_{1}(D_{n,t}E_{n,t}^{out}+F_{n,t}G_{n,t}^{out}+G_{n,t}H_{n,t}^{out})+\varphi_{0})$; $D_{4,t}=(A_{n,t}E_{n,t}^{out}+B_{n,t}G_{n,t}^{out})p_{e}(\theta_{1}(A_{n,t}E_{n,t}^{out}+B_{n,t}G_{n,t}^{out})+\theta_{0})$.

Lemma: For the function *f*(*x*) = log_*m*_*x*, there is *ε*>0, and for which *x*>*ε*, *f*′(*x*)<1 for $m>e^{\frac{1}{\varepsilon }}$.

Now, for the function *f*(*x*) = log_*m*_*x* with *x* = *D*_*j*,*t*_(*j* = 1,2,3,4). The input of electricity, gas, and heat power in each time slot t is positive, clearly. Hence, $E_{n,t}^{in}$, $G_{n,t}^{in}$, and $H_{n,t}^{in}$ are positive. Therefore, there are small enough *ε*_*j*,*t*_>0 where *D*_*j*,*t*_>*ε*_*j*,*t*_>0. According to the lemma, for $m>\max(e^{\frac{1}{\varepsilon_{j,t}}})$; thus, *f*′(*x*) = 1. According to the mean value theorem of integrals,

$$\begin{array}{l} \log_{m}D_{1}+\log_{m}D_{2}+\log_{m}D_{3}-\log_{m}D_{4}-\log_{m}D_{5}\\ -\log_{m}D_{6}<D_{1}+D_{2}+D_{3}-D_{4}-D_{5}-D_{6} \end{array}$$ (17)

because 0≤λ_1_,λ_2_,λ_3_≤1, $\left(2-\lambda_{i}^{2})\geq 1(i=1,2,3\right)$. Thus,

$$\begin{array}{l} \sum_{t\in T}(\log_{m}D_{1,t}+\log_{m}D_{2}+\log_{m}D_{3}-\log_{m}D_{4}-\log_{m}D_{5}-\log_{m}D_{6}\leq \sum_{I}\sum_{t\in T}\left(U_{n}^{i}({\overset{⌢}{I}}_{n,t}^{out})-U_{n}^{i}(I_{n,t}^{out})\right)\\ \leq \sum_{I}\sum_{t\in T}\sum_{i=1,2,3}\frac{1}{3}(2-\lambda_{i}^{2})({\overset{⌢}{U}}_{n}^{i}I_{n,t}^{out}-U_{n}^{i}I_{n,t}^{out}) \end{array}$$ (18)

Thus, the game (15) among energy consumers is an ordinal potential game, which maintains the following potential function:

$$\begin{array}{l} P(y)=\frac{1}{3}\sum_{n\in N}\sum_{t\in T}\sum_{I}(2-\lambda_{i}^{2})(U_{n}^{e}(E_{n,t}^{out})+U_{n}^{i}(I_{n,t}^{out})-U_{n}^{i}(I_{n,t}^{out}))-\sum_{n\in N}\sum_{t\in T}\log_{m}(\theta_{1}E_{n,t}^{in}+\theta_{0})\\ \;-\sum_{n\in N}\sum_{t\in T}\log_{m}E_{n,t}^{in}-\sum_{n\in N}\sum_{t\in T}\log_{m}G_{n,t}^{in}-\sum_{n\in N}\sum_{t\in T}\log_{m}(\vartheta_{1}G_{n,t}^{in}+\vartheta_{0}) \end{array}$$ (19)

where *m*>0. The Jacobian matrix *F* = ∇^2^*P*(*y*) is negative definite regardless of the large m, and the potential function *P*(*y*) is strictly concave. Thus, the ordinal potential game among energy consumers obtains a unique Nash equilibrium [35]. The optimal solution is as follows,

$$\begin{array}{l} \underset{y}{\max}P(y)\\ s.t.\;Equation(9)-(13) \end{array}$$ (20)

### Stackelberg game among suppliers and consumers

We assume that the leader in the Stackelberg game is the energy company who sets energy prices according to consumers’ consumption. After the energy prices are announced, each consumer adjusts their consumption strategy which acts as an ordinal potential game. In this situation, the energy consumption decisions that consumers make will inherently influence each other. The energy company will update energy prices quickly when they are aware of the equilibrium that the consumers’ game reaches. This paper ignores the time lag between energy companies obtaining information from a cloud platform and reacting. As the Jacobian matrix of the energy company revenue function is negative definite, the function is concave, too. Thus, both P(y) and U(p) are concave [36].

Some novel algorithms with good solution performance are generally adopted to solve the mathematical models [37, 38]. Considering the convergence rate of the algorithm, a gradient projection algorithm was chosen to determine the decision-making of consumers. k denotes the number of iterations in the potential game among energy consumers, and s denotes the number of iterations in the Stackelberg game between the energy company and energy consumers. $y_{n}^{k}=\{y_{n,t}^{k}\},n\in N$ are the strategy profiles of consumer 1 to consumer n in the iteration k and time slot t. $y_{n,t}^{k}$ denotes the strategy of consumer n in time slot *t*. A gradient projection method is designed to update the strategy as follows,

$$y_{n,t}^{k+1}=\pi [y_{n,t}^{k}+\gamma \frac{\partial p(y_{t}^{k})}{\partial p(y_{n,t}^{k})}]$$ (21)

where *π* is the projection onto the feasible set that is confined by Eqs (5A)–(5C) and (13), and *γ* is the step size of iteration. The energy companies update the prices $p_{e,t}^{k}$, $p_{g,t}^{k}$, and $p_{h,t}^{k}$ in each iteration. The energy companies also update the ${p^{\prime }}_{e,t}^{k}$, ${p^{\prime }}_{g,t}^{k}$, and ${p^{\prime }}_{h,t}^{k}$, which are the first derivatives of the cost function, respectively.

First, energy consumer n randomly initializes the energy consumption strategy in time slot t. Then, energy consumer n communicates the strategy with the energy companies, which is described as a Stackelberg game. When $y_{n,t}^{k}$ is determined, the energy provided $E_{n,t}^{k,in}$, $G_{n,t}^{k,in}$, and $H_{n,t}^{k,in}$ update as Eq (12). Energy consumer n is aware the game is an ordinal potential. Considering Eq (21), energy consumer n requires the updated values of $p_{e}^{s}(E_{n,t}^{k,in})$, $p_{g}^{s}(G_{n,t}^{k,in})$, $p_{h}^{s}(H_{n,t}^{k,in})$ to determine $\frac{\partial p(y_{t}^{k})}{\partial y(y_{n,t}^{k})}$. However, energy consumer n does not need the strategy profile of the other energy consumers since $\frac{\partial p(y_{t}^{k})}{\partial y(y_{n,t}^{k})}$ is just related to its strategy profile. Finally, when the constraint in step 11 is satisfied, the algorithm will stop and the results are obtained. As all the consumers possess strictly concave potential functions, the designed algorithm will converge very fast and the optimal point is easy to obtain in simulation [39].

**Algorithm 1** Gradient projection algorithm

1: Initialization: Set *t* = 0, *k* = 0, *s* = 0 and *δ* = 10^-6^. Initialize the strategy profile $y_{n,t}^{k}$ for all energy consumers; Initialize the energy inputs $E_{n,t}^{k,in}$, $G_{n,t}^{k,in}$, and $H_{n,t}^{k,in}$ according to Eq (12); Initialize the energy prices $p_{e}(E_{n,t}^{k,in})$, $p_{g}(G_{n,t}^{k,in})$, $p_{h}^{s}(H_{n,t}^{k,in})$ according to Eq (5A)–(5C).

2: For *t* = {1,2,⋯,*T*}

3:    For *n* = {1,2,⋯,*N*}

4:        Update $y_{n,t}^{k+1}$ according to Eq (21).

5:        Update $E_{n,t}^{k,in}$, $G_{n,t}^{k,in}$, and $H_{n,t}^{k,in}$ according to Eq (12).

6:    End

7:    *k*←*k*+1

8:    Until $\left\|y_{n}^{k+1}(t)-y_{n}^{k}(t)\right\|<\delta$

9:    Update $p_{e}^{s}(E_{n,t}^{k,in})$, $p_{g}^{s}(G_{n,t}^{k,in})$, $p_{h}^{s}(H_{n,t}^{k,in})$ according to Eq (5A)-(5C).

10:    *s*←*s*+1

11:    Until $\left\|p_{i}^{s+1}(I_{n,t}^{k,in})-p_{i}^{s}(I_{n,t}^{k,in})\right\|<\delta ,I=\{E,G,H\}$

12: End

## Simulation

This section will evaluate the performance of the proposed gradient projection algorithm. There are one company and ten consumers in the system. T is divided into 24 time slots. The efficiency parameters *η*_*ge*_, *η*_*gh*_, and *η*_*eh*_ are selected randomly from [0.4, 0.5], [0.35, 0.45], and [0.4, 0.5], respectively. The electricity gas and heat power generation cost function has coefficients *θ*_1_ = 3, *θ* = 3, *ϑ*_1_ = 2, *ϑ* = 0.1, *φ*_1_ = 1, and *φ* = 4. The coefficients $w_{n}^{e}$, $w_{n}^{g}$, and $w_{n}^{h}$ were selected randomly from [10, 20] in time slots 11–14, and [15, 25] from 16:00 to 21:00. The parameters are chosen from [5, 10] in other time slots. $\alpha_{n}^{e}$, $\alpha_{n}^{g}$, and $\alpha_{n}^{h}$ are set to be 0.5 for every energy consumer. The dispatch factors *λ*_1_, *λ*_2_, and *λ*_3_ were set to be 0.7, 0.1, and 0.2.

**Fig 2:**
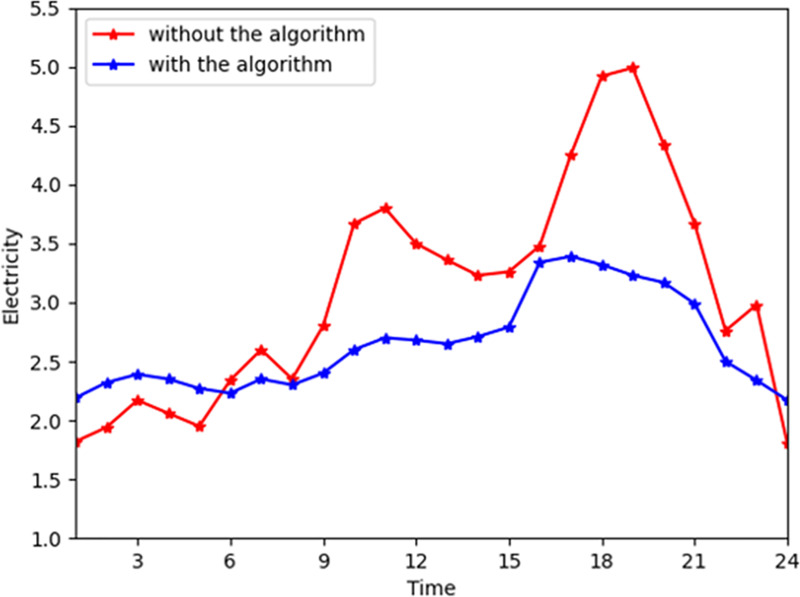
The total electrical power from the company with and without the algorithm.

**Fig 3:**
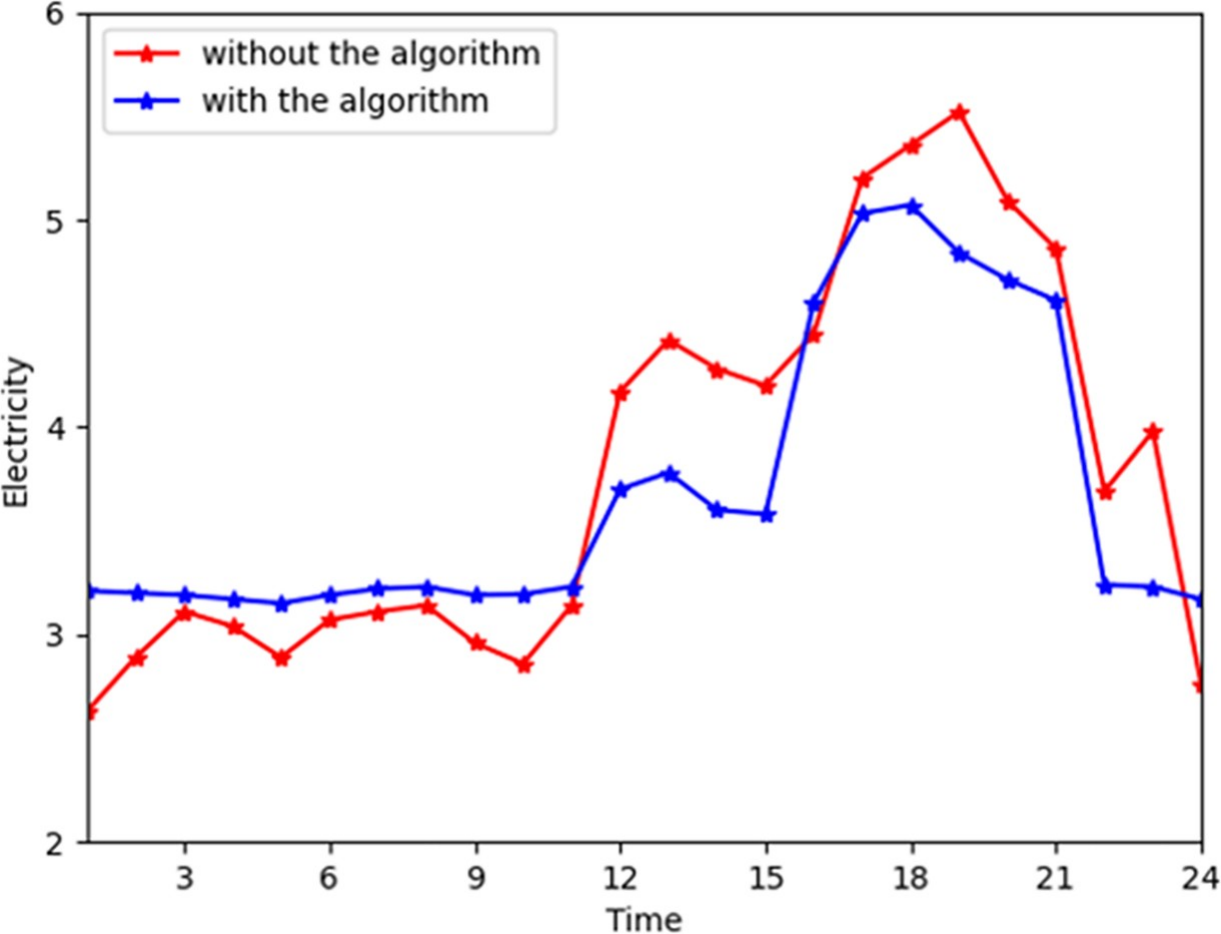
The total electrical power consumption with and without the algorithm.

Fig 2 shows the general change trend of electrical power with and without the algorithm. In the MCS, the energy company generates electrical power directly. The company reduces the generation cost by decreasing the electrical power peaks. Without the algorithm, the electrical peaks are during time slots 16 to 21. By contrast, the peaks are reduced apparently, reaching about 40% with the algorithm. Fig 3 shows the general change trend of electricity consumed by the customers. With the algorithm, the peaks only drop by approximately 10%. This is because the customers prefer the instant satisfaction of the need for energy consumption and have no need to reduce the cost by shifting their demand from peak hours to off-peak hours. This has a slight impact on both energy consumers and companies. For the coefficients *α* of the customers' satisfaction functions are higher, the peak during time slots 16 to 21 in Fig 3 is higher, which means the customers maintain current their electricity consumption pattern.

**Fig 4:**
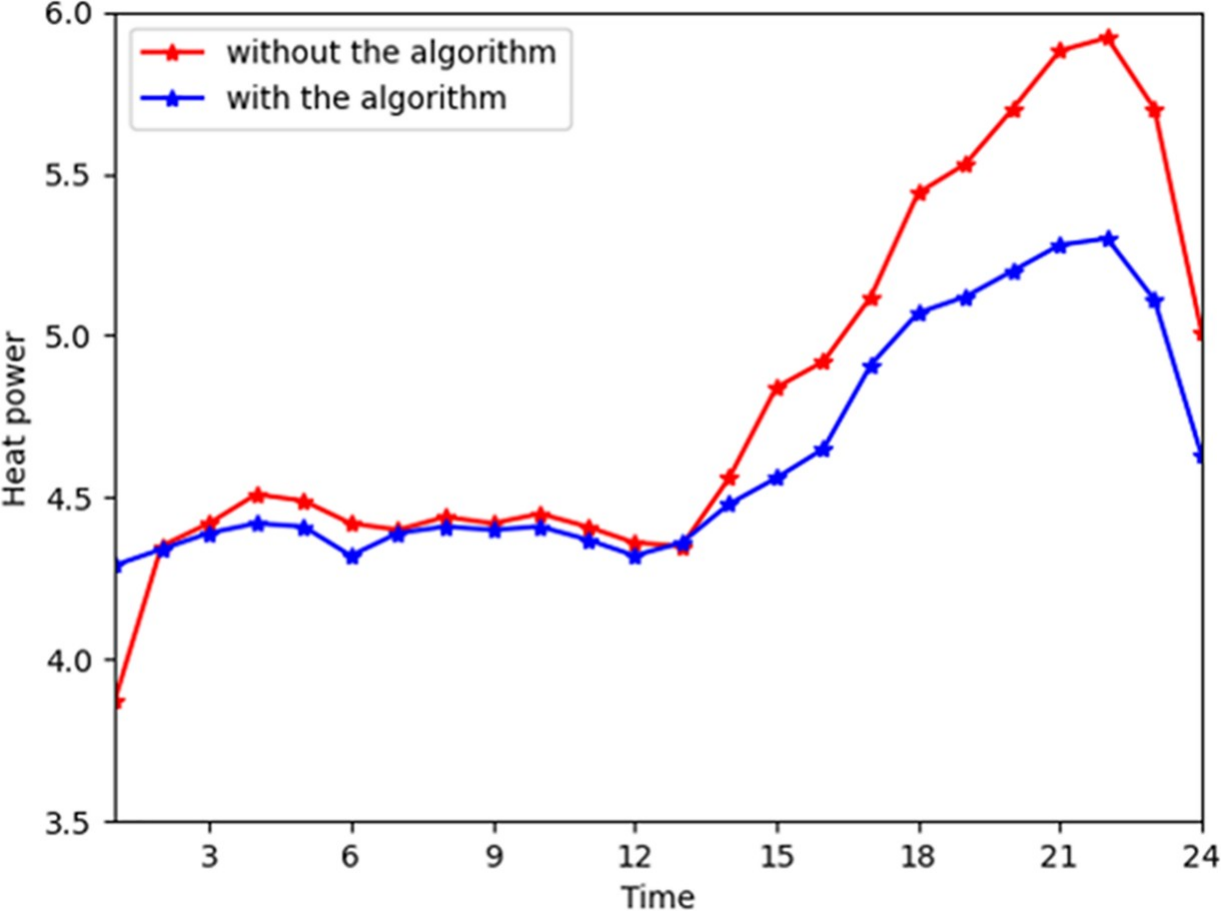
The total heat power from the company with and without the algorithm.

**Fig 5:**
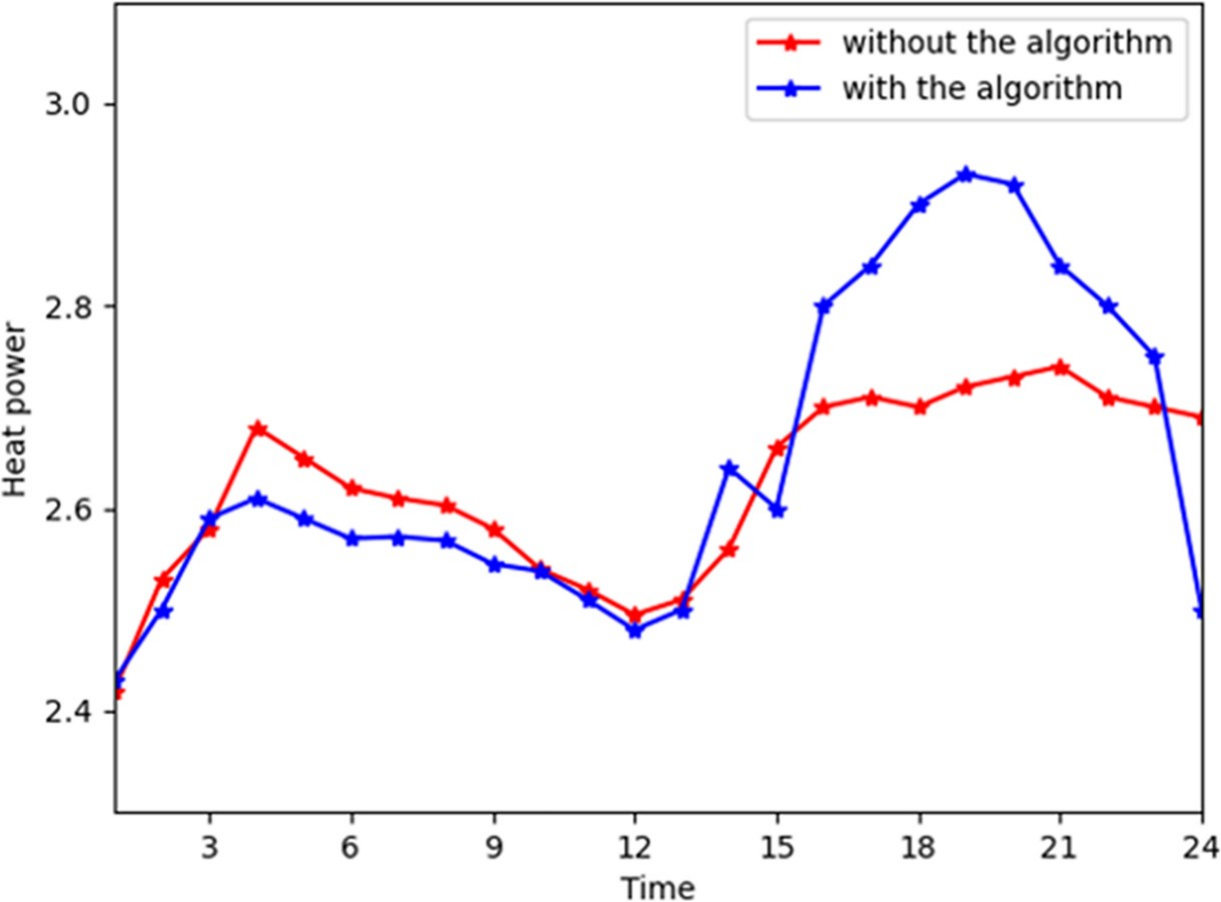
The total heat power consumption with and without the algorithm.

Fig 4 shows the general change trend of heat power purchased from the energy company. The energy company profile soars at time slots 15 to 21. In reality, during time slots 15 to 21, the energy company provides more heat power to consumers due to the increased demand. During time slots 15 to 21, consumers convert their consumption patterns from heat power to electricity and gas transformation consumption. Thus, the heat power provided by the energy company deceased in the industrial interconnection. Fig 5 shows the general trend of heat consumption on the customer side. The peaks in industrial interconnection are even increased by 7% during time slots 15 to 21 because the customers' satisfaction functions are higher in these hours. In Fig 3, the peak of the electricity reduces due to shifting from electrical power to heat power. Thus, the consumers will purchase less heat power and more electricity and gas to shift the consumption patterns. More gas and electricity are input in the converter device to convert to heat power due to the high satisfaction parameter of consuming heat power.

**Fig 6:**
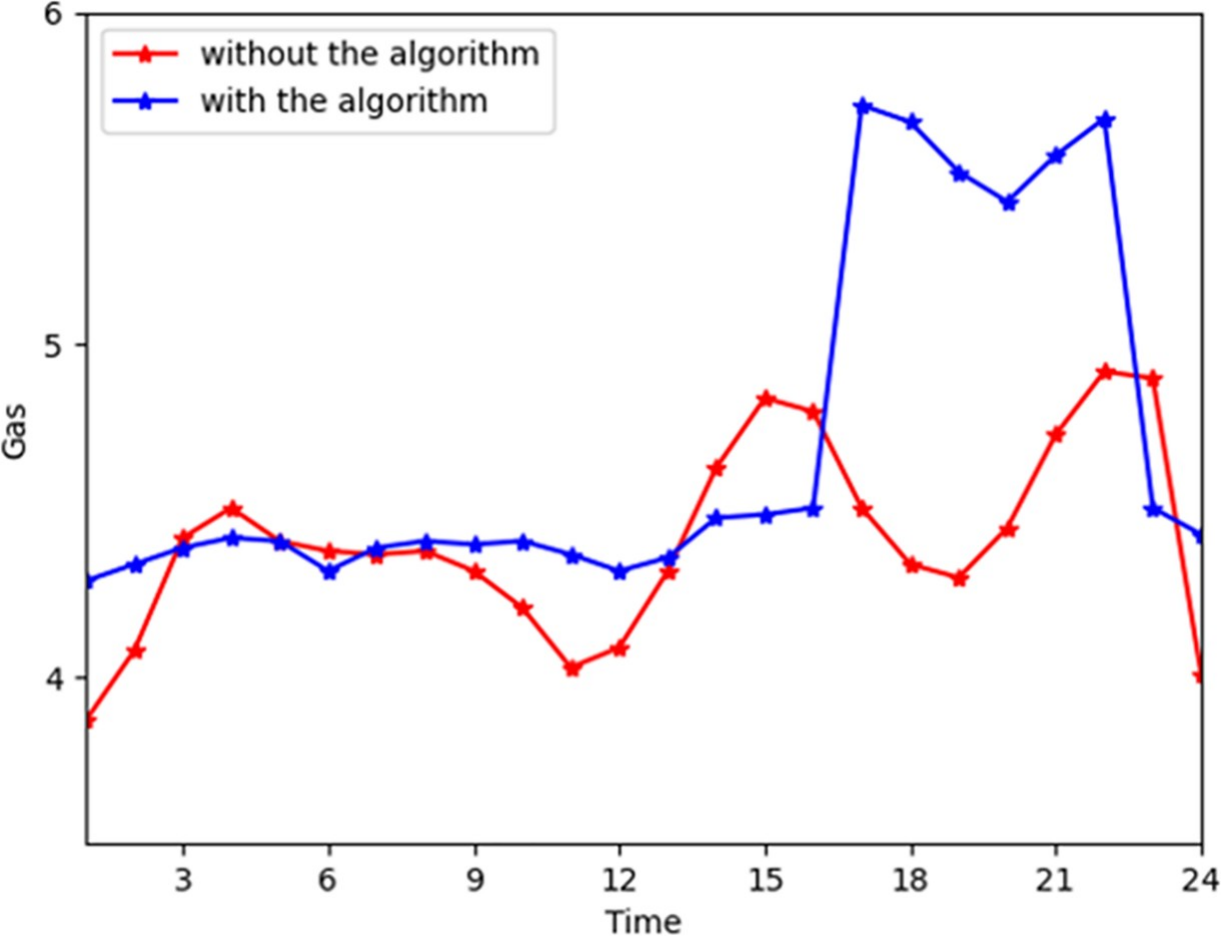
The total gas from the company with and without the algorithm.

**Fig 7:**
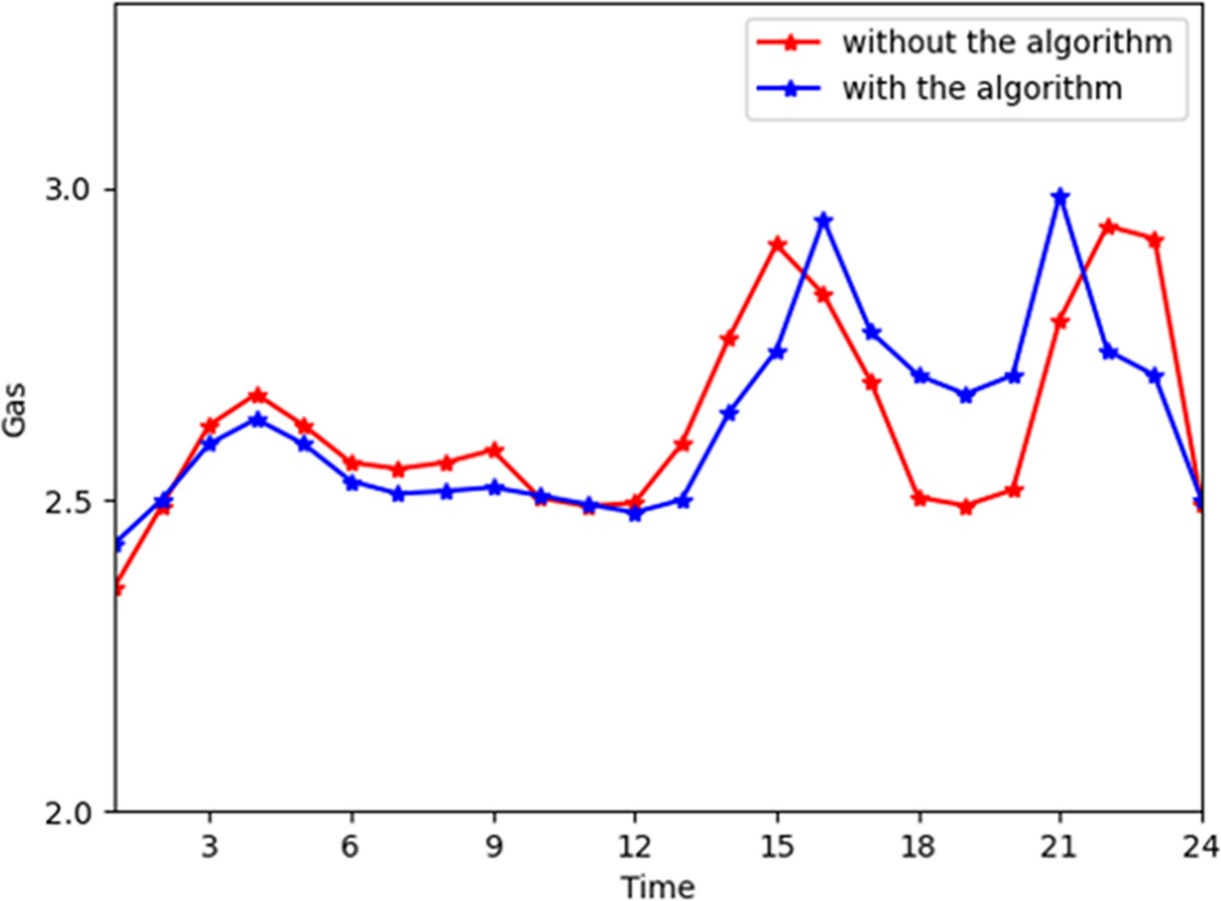
The total gas consumption with and without the algorithm.

Fig 6 shows the general change trend of gas power provided by the energy company. In the industrial interconnection, the gas of the energy company is flattened during time slots 0–16 and 21–24 and decreases significantly during time slots 16–21. During time slots 16–21, the consumers require more gas and less electricity and heat power. Thus, the cloud platform directs the device to stop converting. Fig 7 shows the gas general consumption trend on the customer side. As the customers' satisfaction functions are higher during time slots 16 to 21, the peaks of gas consumption are increased slightly. The gas consumption is approximately 2.65 on average. However, the average gas provided by the energy company is approximately 4.4. The reason can be that the device converts more gas to electricity and heat power. According to Figs 3 and 5, the demand for switching electricity to heat power is increased, and the demand for electricity is decreased. Thus, the energy company provides more electricity and gas to consumers and the consumers will consume more gas and less electricity and heat power than the actual demand.

**Fig 8:**
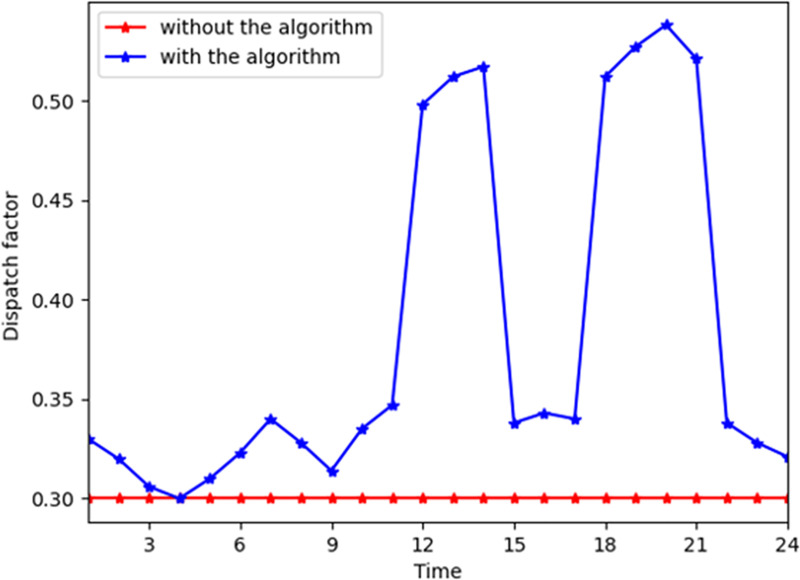
Parameter *λ*_1_ at different time slots for a consumer with and without the algorithm.

**Fig 9:**
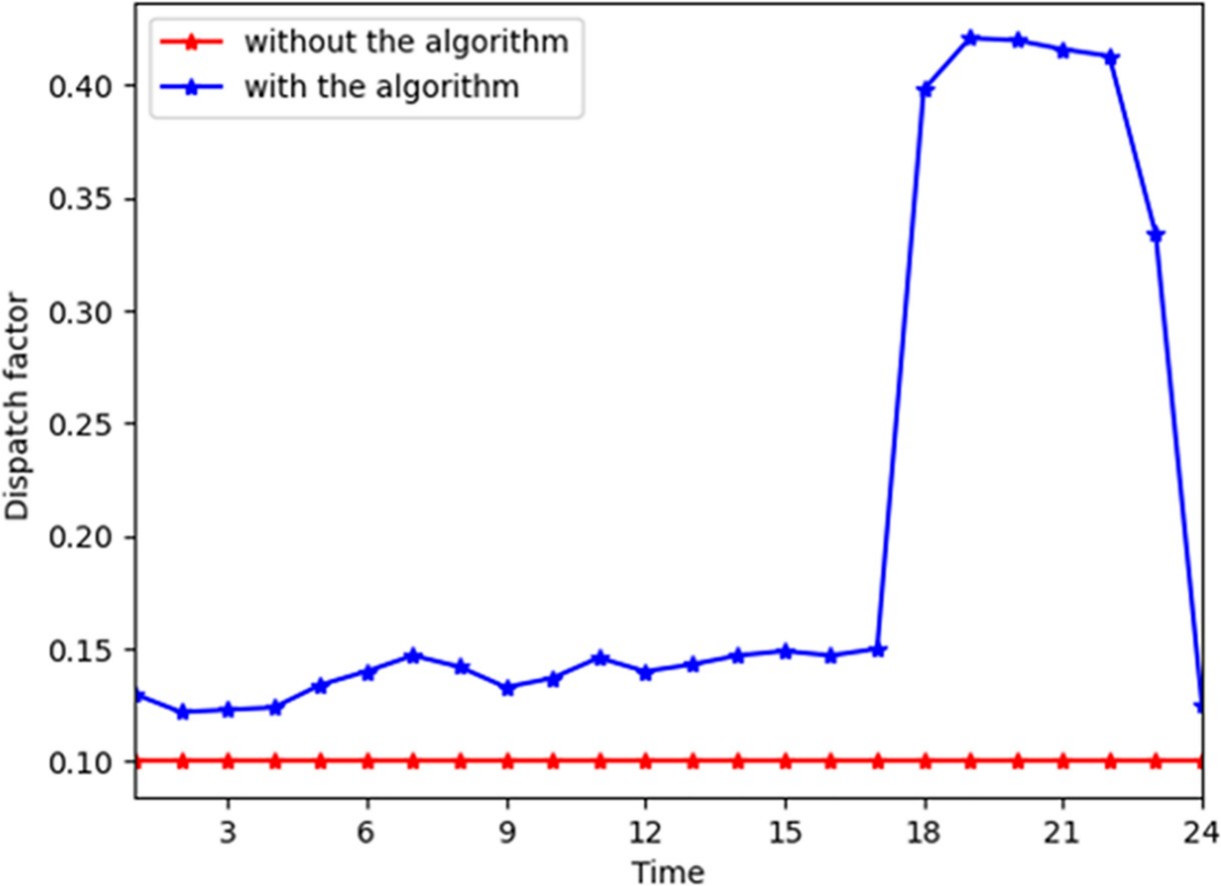
Parameter *λ*_2_ at different time slots for a consumer with and without the algorithm.

**Fig 10:**
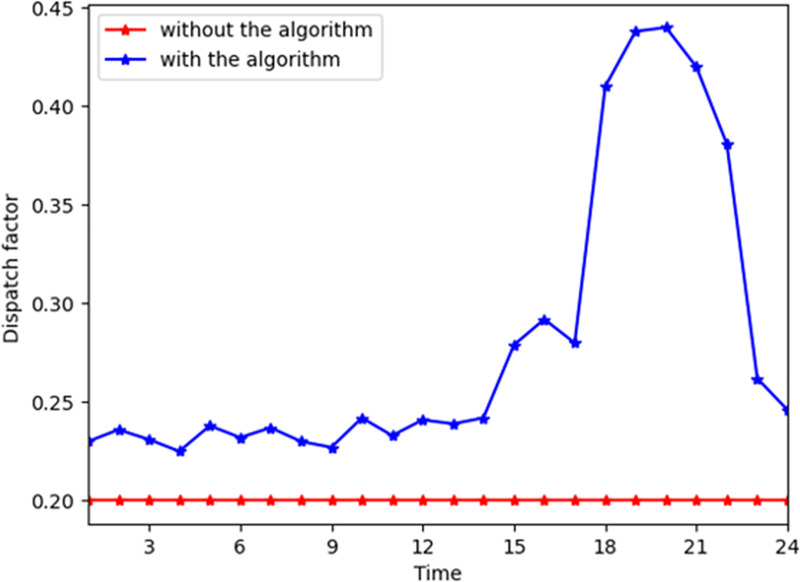
Parameter *λ*_3_ at different time slots for a consumer with and without the algorithm.

Fig 8 shows the influence of parameter *λ*_1_ for consumer n. In the MCS, *λ*_1_ becomes higher at all time slots generally and lower during time slot 20–24. This indicates that the energy resource of gas is converted to electricity during that time. Similarly, Figs 9 and 10 depict that *λ*_2_ and *λ*_3_ become higher when electricity and gas provided by the energy company are converted to heat power through the converter device, respectively. *λ*_1_ reaches its maximum during time slots 11–14 and 16–21 when the demand for electricity increases on the consumers’ side. *λ*_2_ and *λ*_3_ reach their maximum during time slots 16–21, which indicates that the customer demand is higher in these time slots. Thus, they prefer to shift the energy consumption pattern by switching the types of energy demand in this time slot.

**Fig 11:**
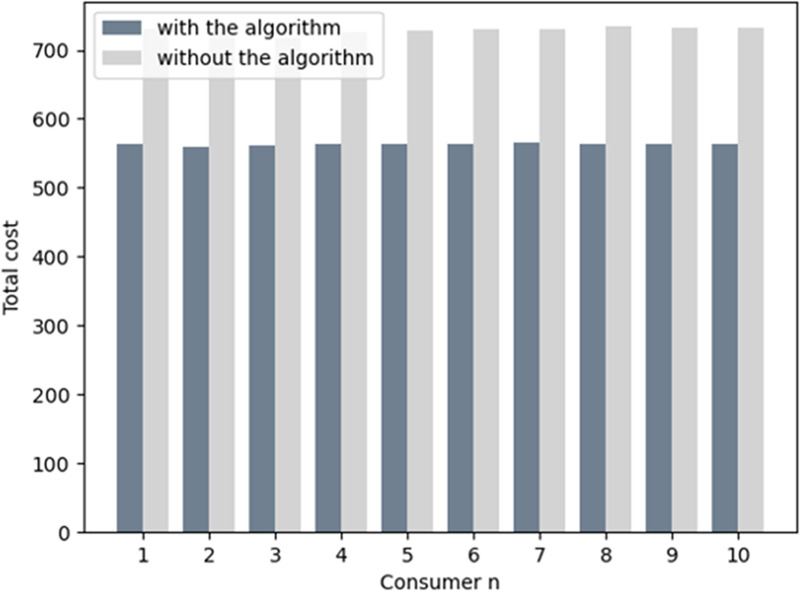
The total cost for consumers with and without the algorithm.

**Fig 12:**
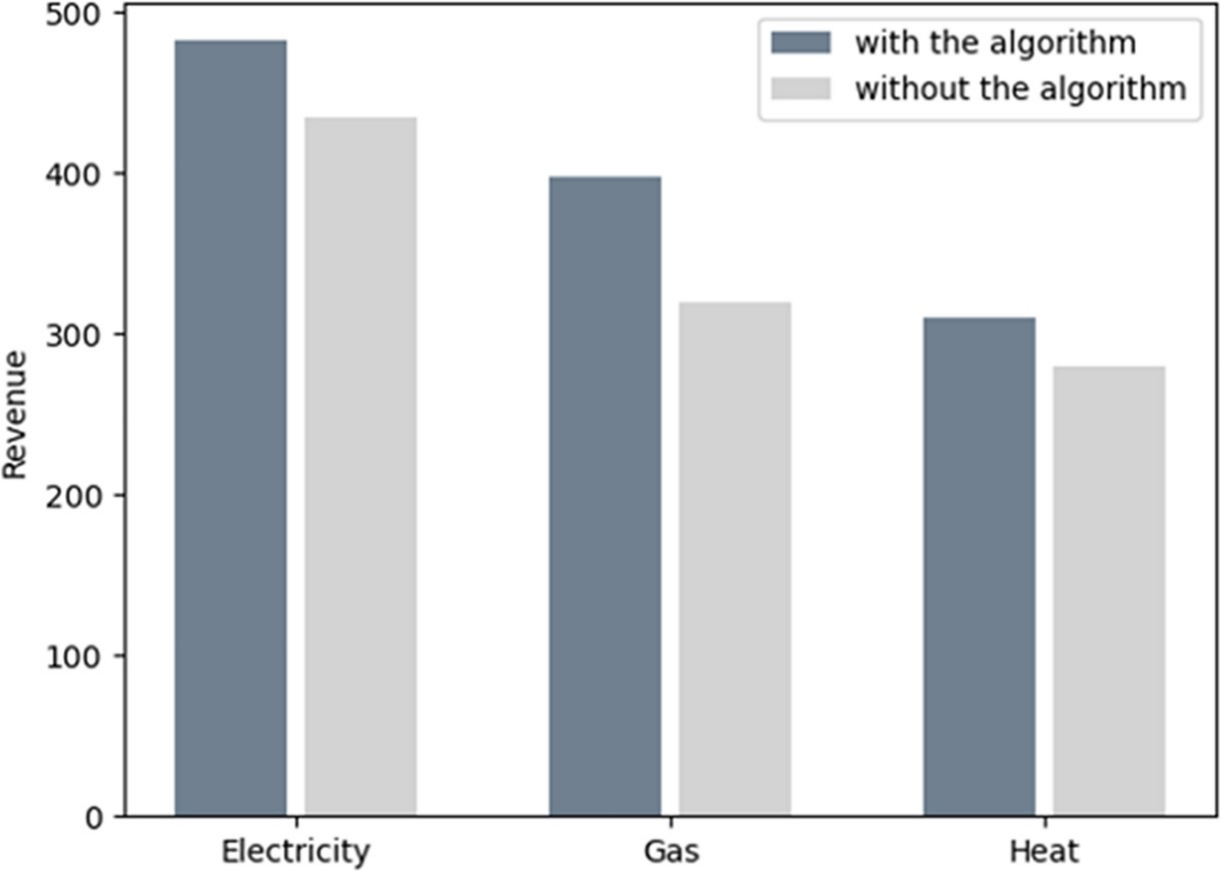
The revenue for providers with and without the algorithm.

Fig 11 represents the total cost which is the sum of the electricity bills, gas bills, and heat power bills for all consumers. The daily bill of each consumer is reduced by about 35% by participating in the MCS. Hence, by reducing the cost, the system coupled with a cloud platform benefits both the customers and energy companies. Fig 12 shows the profit of every energy company with and without an algorithm in MCS. The profit of the energy company clearly increases in industrial interconnection as the energy consumption pattern is transformed. Thus, the cost decreases for the energy company. In Fig 12, the revenue of electricity, gas, and heat power provided by the energy company increase, which denotes that the consumers require more gas than electricity and heat power during peak hours. Hence, the revenue by providing gas is greater than the other two sources of energy.

**Fig 13:**
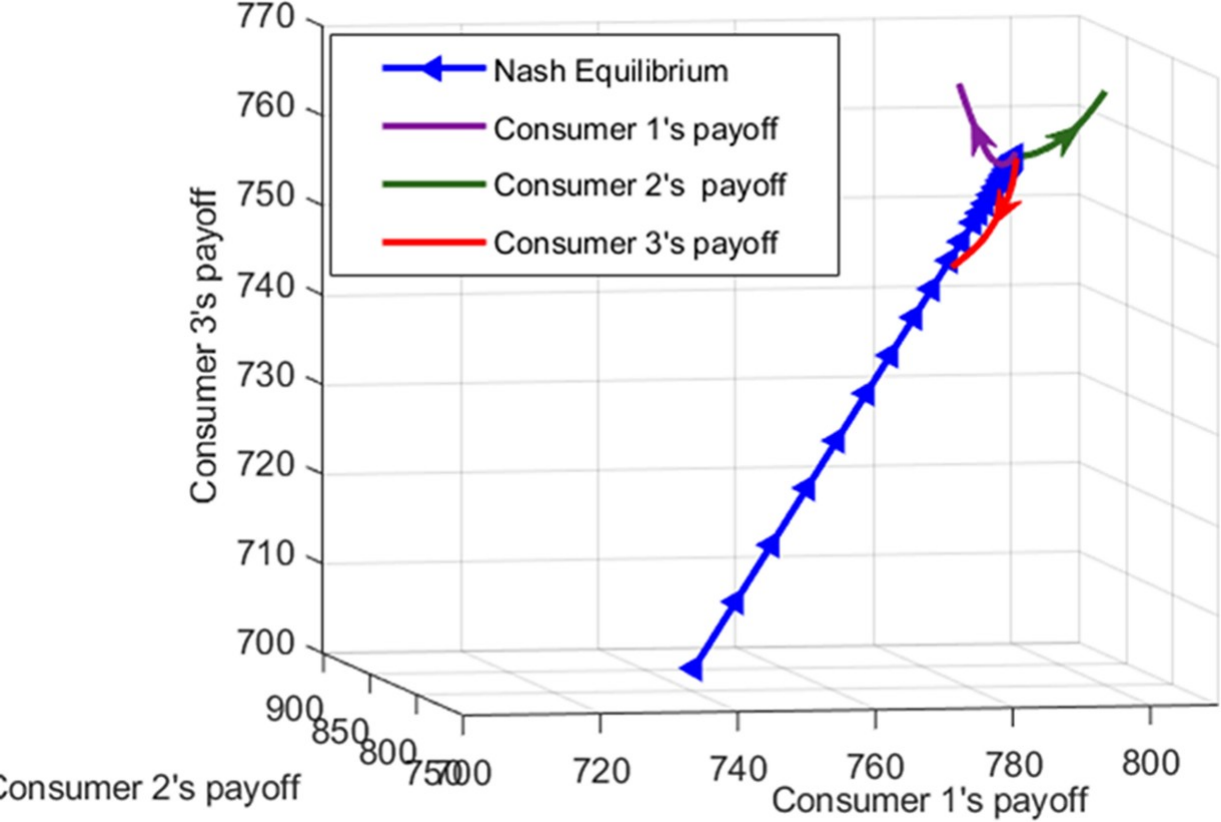
The convergence to the Nash equilibrium.

**Fig 14:**
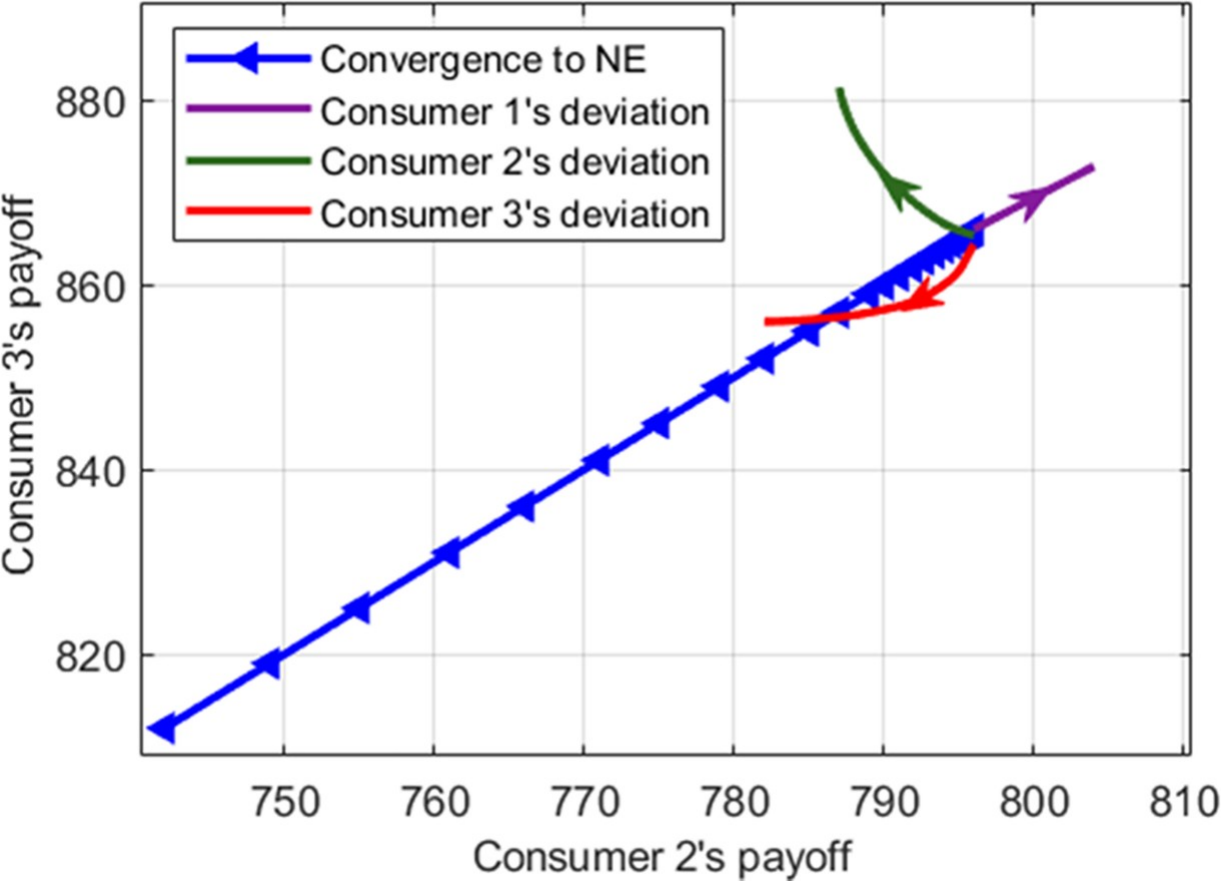
The impact of deviation on the consumers' payoff.

In order to clarify the convergence of the proposed algorithm, the number of consumers is set as three to investigate the strategy profiles in the equilibrium. Figs 13 and 14 show the convergence of the payoff for consumers 1, 2, and 3 to the Nash equilibrium in two ways. Apparently, the payoff of every consumer approaches the Nash equilibrium point in all iterations. The green, purple, and red curves with arrows show the change of payoff when the consumer's choice deviates from the Nash equilibrium strategy profile unilaterally. For example, Consumer 1 chooses to deviate from its equilibrium strategy profile while the strategies of other consumers are considered unchanged. Thus, consumer 1 changes the dispatch factor during peak hours to consume less gas and more electricity and heat power. This shows that the payoff of consumer 1 is gradually increasing in Figs 13 and 14. However, the payoff of consumer 3 decreases when the consumer deviates unilaterally. In fact, when consumer 1 changes the dispatch factor, the consumption patterns conversion leads to an increase in the amount of gas converted into electricity and heat power as well as a rise in the price of gas. At the same time, the more electricity and heat power the converter generates during this time, the less it buys from the energy company. As a result, the prices of electricity and heat power fall, and the payoffs of the consumer 2 and 3 will increase. Consequently, neither consumer is inclined to deviate from the selected equilibrium strategy profile in the Nash equilibrium.

## Conclusions

In this paper, we present a multi-energy conversion system coupled with a cloud platform. Utilizing effective metering causes customers to manage their electricity, gas, and heat power consumption. We then propose a novel practical integrated consumption for energy consumers. The complex energy consumption pattern is described as an ordinal potential game, the purpose of which is to digitize the competition and cooperation of consumers of energy companies. We prove that the Stackelberg game and ordinal potential game both have unique Nash equilibriums. A gradient projection algorithm is also proposed to solve those two games. Lastly, simulations are performed to evaluate the performance of the algorithm. This demonstrates that an energy system with one energy company and ten consumers improves the efficiency of energy conversion through the cloud platform and made electricity, gas, and heat power networks steadier. The cost of energy companies and consumers is also decreased after implementing the cloud platform, leading to the development of new businesses with more profit.

With the increasing awareness of environmental protection, the coordinated development of energy, environment, and economy has become an urgent need for the development of China's digital economy. Multi-energy conversion and complementarity can combine various energy sources to alleviate the contradiction between energy supply and demand, promoting a virtuous cycle of the ecological environment through extensive use of renewable energy. The integrated system with energy conversion as the core adopts advanced information technology to integrate multiple energy inputs, multiple product outputs, and multiple energy conversion units. Especially with the rapid development of renewable energy, its consumption and transportation of renewable energy is of great significance and has contributed to the realization of China's energy transition.

## Supporting information

S1 Data (XLSX)
